# Reproductive carrier screening: perspectives for a future implementation in Germany

**DOI:** 10.1515/medgen-2026-3032

**Published:** 2026-11-06

**Authors:** Sabine Rudnik-Schöneborn, Klaus Zerres, Nicole Sänger, Ursula Eichenlaub-Ritter, Simone Heidemann

**Affiliations:** Institute of Human Genetics Medical University of Innsbruck Peter-Mayr-Straße 1 6020 Innsbruck Austria; RWTH Aachen University Hospital Center for Human Genetics and Genomic Medicine Pauwelsstr. 30 52074 Aachen Germany; University Hospital Bonn Department of Gynecological Endocrinology and Reproductive Medicine, VenusKind at UKB Venusberg-Campus 1 53127 Bonn Germany; Bielefeld University Department of Biology Universitätsstr. 25 33615 Bielefeld Germany; Institute for Tumor Genetics North Fraunhoferstr. 16 24118 Kiel Germany

**Keywords:** Reproductive carrier screening, perspectives, health care system, reproductive options

## Abstract

Reproductive carrier screening (RCS) has made its way into the health care system in various countries. Current data suggest that 3.5 % of European couples are carriers for the same autosomal recessive disease and an additional 1.0 % of women are carriers of an X-linked disease. Once an increased genetic risk has been identified, couples can choose between a range of options, including prenatal or preimplantation diagnostics. Also alternatives apply such as gamete donation, reconsidering their desire to have children, adoption, or accepting a child with the condition.

Improved reproductive autonomy is the main argument for RCS along with the concept of the best possible personalized medicine according to the current state of science and technology. The individual desire of couples to determine genetic risks for future children should be acknowledged and politically legalized. The authors outline the legal, genetic and reproductive, and ethical perspectives for RCS in the German healthcare system.

## Introduction

The term “reproductive carrier screening” (abbreviated as RCS) is often referred to as “preconception carrier screening” or “expanded carrier screening.” It refers to genetic testing of a couple before or during pregnancy to identify a high risk situation for a child with a serious genetic disorder. To emphasize that this is not just an offer *before* pregnancy (preconception) and not a global expansion (“expanded” of what?) of screening, the authors of this article have opted for the term reproductive carrier screening.

In contrast to parents of an affected child who wish to take reproductive measures for further pregnancies and know what diagnosis or issue is involved, the situation is different in the case of a non-targeted search (screening) for mutations. These are couples who at the time of testing are not considered to be at risk for a particular disease. This means that there are special requirements regarding the scope of the tests offered and the conditions for informed and considered decisions when there is a desire to have children.

Naturally, RCS does not allow to clarify all future risks for genetic diseases or malformations in children. The concept is that only couples are identified who have an increased risk for ***autosomal recessive (AR) or X-linked (XL) disorders***. If both parents carry a heterozygous mutation in the same AR gene, the probability of a child developing the disease – regardless of sex – is 25 %. If the mother is a carrier of a recessive XL disorder, future sons have a 50 % risk of developing the disease. RCS cannot rule out chromosomal copy number variants or new mutations or mutations in the mitochondrial DNA of a future child. Moreover, the basic risk of congenital malformations – mostly based on multifactorial causes – is around 3 %.

With the advancement of molecular genetic methods, RCS has become more precise and concrete in recent years. The feasibility and acceptance of RCS was examined in detail in a large Australian study (Mackenzie’s Mission) involving more than 9,000 couples [1]. Enrollment took part via health care providers who provided potential participants with a study invitation and individual access to an online portal. Of the more than 19,000 invited couples 10,038 enrolled in the study (uptake rate of 45.9 %), and 9,107 (90.7 %) underwent genetic testing. An increased risk for children (‘increased chance’) was only reported if *both partners* carried a pathogenic or likely pathogenic variant in the same gene for an AR disease or if a corresponding variant was detected in a gene for an XL disease *in the female partner*. No variants of uncertain significance (VUS) or AR carrier status in either partner were reported. Couples with an increased risk were contacted by genetic counsellors and informed about the result and its consequences for reproductive options. As part of the study, couples with an increased risk were offered the opportunity to undergo a cycle of preimplantation genetic diagnosis free of charge.

In 175 (1.9 %) couples, a risk situation previously unknown in the family was discovered, while in a further 180 (2.0 %) couples, an increased genetic risk was already known. In cases of existing pregnancy, 29 out of 45 (64 %) couples opted for prenatal diagnosis. Of 130 couples wishing to have children, 95 (73.1 %) opted for pre-implantation genetic diagnosis. Anxiety and health related quality of life was studied at enrolment, sample collection, and 3 months after receiving the test result. Trait anxiety was rated as low or moderate and increased only in couples with “increased chance” of having a child with a genetic condition. Quality of life scores were high at enrolment and remained stable after testing. 98.9 % of participants perceived screening to be acceptable [1].

Overall, Mackenzie’s mission is considered a great success, as stated in the foreword to the NEJM [2]. Decisive arguments for the high uptake of the RCS were seen in the study design, the involvement of key stakeholders, free access to a broad gene panel for all couples wishing to have children – including the option of pre-implantation genetic diagnosis – and good accompanying research. Meanwhile Mackenzie’s mission has been completed and RCS is reduced to a Medicare bulk-billed standard testing by the Australian Government. Since 2023, all women can undergo RCS for cystic fibrosis, spinal muscular atrophy, and Fragile X syndrome (https://www9.health.gov.au/mbs/fullDisplay.cfm?type=item&q=73451, access 30 June 2026).

## Recommendations of academic societies

In German-speaking countries, there is a political restraint regarding RCS, most likely in the context of their history with eugenics in Nazi Germany. No recommendations exist specifically in terms of RCS. The German Society for Human Genetics (GfH) published a statement at the end of 2023 about incidental genetic findings in exome or genome studies which includes carrier status as optional group 3 findings regardless of the age of the analysed individual [3]. The argument is that parents should be given the opportunity to be informed of such incidental findings identified in minors so that they can make informed decisions regarding family planning.

The European Society of Human Genetics (ESHG) [4] first released recommendations for so-called expanded carrier screening (ECS) in 2016. The argument in favour of offering ECS is the reproductive autonomy of couples who wish to have children and who can choose from *different courses of action,* if an increased risk of a serious disease in future children is detected. While the ESHG guideline points out that general consent (“generic consent”) of the couple is sufficient prior to ECS, an increased risk for the couple’s children is a situation that must be explained in the context of specialist genetic counselling. Depending on the legal basis in the respective countries, the options for action are primarily the offer of prenatal diagnostics or preimplantation diagnostics, but also alternatives such as egg or sperm donation, reconsidering the desire to have children, adoption, or the acceptance of a potentially affected child, including the associated preventive care.

In 2013, the American College of Medical Genetics (ACMG) advocated for general (pan-ethnic) carrier screening for cystic fibrosis and spinal muscular atrophy, supplemented by ethnicity-specific screening for Ashkenazi Jews [5]. This was similarly recommended in 2017 by the American College of Obstetricians and Gynecologists (ACOG), supplemented by laboratory chemical analyses in women to clarify the risk of hemoglobinopathy and an analysis for *FMR1* premutations in cases of suspected fragile X syndrome carrier status [6].

In its current recommendation, the ACMG proposes a four-tiered approach [7] that considers the frequency of the respective diseases in the American population and the origin or consanguinity of the couple. The first tier (Tier 1) covers risks for cystic fibrosis and spinal muscular atrophy (the most common AR diseases in the US), while the second tier (Tier 2) covers all genes for AR diseases with a carrier probability of at least 1 % (>1 in 100). In the third tier, all AR diseases with a carrier probability of at least 0.5 % (>1 in 200) and XL disorders with a frequency of at least 1 in 40,000 are to be tested. In the fourth stage (Tier 4), all AR or XL disorders should be tested via exome analysis. The general recommendation is to offer Tier 3 analysis as a matter of principle, and Tier 4 analysis only for consanguineous couples or those with specific questions arising from their personal and family history.

Recently, the Canadian College of Medical Geneticists has published a position statement and recommends a pan-ethnic RCS via a provincial or territorial programme for a limited panel of relatively common and severe childhood onset genetic conditions. These are based on Canadian experience: cystic fibrosis, fragile X syndrome, spinal muscular atrophy, hemoglobinopathies, and founder mutations for Tay-Sachs disease, Canavan disease and familial dysautonomia. However, the working group does not recommend publicly funded population-level expanded carrier screening currently [8].

## The legal perspective in Germany

Genetic diagnostics for medical purposes and genetic counselling have been regulated in Germany since 2010 by the Genetic Diagnostics Act (GenDG). Genetic testing with the aim to clarify a predisposition to diseases or health disorders in offspring is defined therein as predictive genetic testing (§ 3 No. 8b GenDG), for which the legislator requires genetic counselling by a physician qualified for genetic counselling prior to the analysis and after the test results are available.

Despite using the term “screening” in RCS, this does not imply that it should be implemented as a genetic screening programme in a legal sense. A “Genetische Reihenuntersuchung” according to § 3 (9) GenDG is a genetic test carried out systematically on the general population or specific groups of people, without any further eligibility criteria (Explanatory Memorandum to the Genetic Diagnosis Act (GenDG), BT-Drs 16/10532, 22). In contrast, an RCS is legally permissible as an individual genetic test on a case-by-case basis both in the case of a conspicuous family history and independently of any specific circumstances.

According to § 7 (1), an RCS may only be initiated as a predictive genetic analysis by specialists in human genetics and specialists in gynaecology and obstetrics (§ 7 (1) GenDG). Specialists in gynaecology and obstetrics need to qualify for genetic counselling, if they themselves carry out the genetic counselling required by law before and after the RCS. Otherwise, they can also refer those seeking advice to other physicians who are demonstrably qualified to provide genetic counselling.

## The genetic and reproductive perspectives

### Which diseases should be tested for?

If a decision is made to offer RCS, a healthcare system needs clear regulations for the selection of genes to be examined, the use of genetic analysis methods, and the clinical validity of mutations tested. It is beyond the scope of this overview to go into detail here on the question of testing genetic variants for pathogenicity and classifying the severity of a disease included in the test panel. Following the ESHG recommendations [4], genetic carrier screening should primarily focus on serious, congenital and childhood diseases. However, the seriousness of a condition is a complex and multi-layered concept which is still ill-defined [9]. In Australia, the attitudes of adults without a personal or familial history were investigated which conditions with different ranges of severities should be included in RCS. Prospective parents wanted a tiered approach to RCS and the ability to choose the severity of conditions included in screening [10].

### What are the expected detection rates of high-risk couples?

According to calculations by Hotakainen et al. [11], the carrier frequency is at least 1 in 200 for a total of 100 genes. With this cut-off, the authors calculate that *3.5 % of couples in the Non Finnish Europeans (NFE) population have an increased risk of an autosomal recessive disease and 1.0 % of couples also have a risk of an X-linked disease* in their children. This proportion of positive couples is therefore significantly higher than in mixed populations (e.g. in the US or Australia), where the proportion of high-risk couples is assumed to be 1–2 % [12].

The systematic analyses of over 700,000 exome data gnomAD v4.1.0 for calculating carrier frequencies in different populations are similarly complex [13]. According to this study [13] only 78 AR and XL genes in NFE Europeans have a carrier frequency of at least 1 in 200 (analogous to Tier 3 of the ACMG recommendations). The probability of couples with increased genetic risk is given here as approximately 3 %.

Carrier frequencies are of course influenced by migrant populations in German-speaking countries, and genetic risk to children rapidly increases in consanguineous couples [14].

### How can the target group, i.e. couples who want to have children or are already pregnant, best be reached?

In any case, it is desirable to give couples the opportunity to obtain information about an increased risk *before* pregnancy. In the German healthcare system, first contact can best be provided by family doctors or gynaecologists. For those couples who express interest in RCS a formal genetic counselling is required.

In studies in the Netherlands and Australia, healthcare professionals were trained to inform, educate, and support couples about RCS. In the Netherlands, family doctors were trained for this purpose [15]. In Australia, various *healthcare professions* (doctors, genetic counselors, midwives, nurses) were involved in providing information and education [1].

### Which tools are suited best for providing information and obtaining informed consent for RCS?

The selection of materials that allow an informed decision for or against the test is of great importance in the age of social media and should be examined in scientific studies for the German-speaking world. An American study compared various sources of information (brochure, video, or comic) for RCS. The comic book group performed best in terms of knowledge gained about the test, while there was no difference between the groups in terms of decision-making and potential conflicts [16]. With the help of artificial intelligence, additional information materials could also be developed as interactive platforms that would allow frequently asked questions from couples wishing to have children to be answered in advance.

### What do people expect from RCS and what concerns do they have in connection with such an offer?

In the context of accompanying research on the benefits and risks of RCS in the Netherlands [17], the arguments in favour of using it included the avoidance of possible suffering and death in a child, better preparation for future pregnancies, knowledge of genetic characteristics, and the prediction of psychosocial and economic consequences for the family.

Reasons against using RCS are primarily seen in the emotional strain on the relationship and the family when an increased genetic risk is detected, feelings of guilt when a disabled child is born, possible social pressure to avoid having children with disabilities, concerns about one’s own health risks, or the stigmatization or discrimination of carriers. Previous studies from the Netherlands and Mackenzie’s mission have shown that RCS does not have a negative psychological impact.

### How should abnormal findings be communicated?

According to the ESHG recommendation – as also implemented in Australia – *couple-based communication of risks* should take place, as a heterozygous carrier status can be expected in *every single person of European descent* with a probability of at least 50 % based on an exome data comprising 415 AR genes [18]. All feedback on abnormal findings must be provided by a specialist in human genetics. Depending on the frequency of a disease in the population and individual requests the disclosure of a carrier status might result in cascade screening for relatives who wish to have children.

## What are the reproductive options for couples with an increased risk?

Bearing in mind that reproductive medicine in Germany continues to be governed by a 33-year-old Embryo Protection Act (https://www.gesetze-im-internet.de/ eschg/BJNR027460990.html) which restricts couples’ reproductive autonomy and accepts health risks to mother and child, there are some important legal principles in the field of reproductive medicine that should be modernised.

Once pregnancy has been confirmed, non-invasive and invasive options are available to determine a fetal genetic risk for chromosomal anomalies. When the non-invasive prenatal test (NIPT) using cell-free DNA in maternal blood was authorised for the detection of trisomy 13, 18 or 21, it was noted that the test is also beneficial even in the absence of any abnormal preliminary findings, for example from first-trimester screening. According to the wording of the Joint Federal Committee (G-BA), “a non-invasive prenatal test (NIPT) for trisomies 13, 18 and 21 be carried out at the expense of the statutory health insurance scheme if, in the course of medical antenatal care, the question arises as to whether a fetal trisomy might be present, and the uncertainty constitutes an unreasonable burden for the pregnant woman.” (https://www.g-ba.de/themen/methodenbewertung/schwangerschaft-mutterschaft/faqs-nipt/ accessed 30 June 2026). This clearly shows that NIPT as a service covered by health insurance no longer requires any real justification, and that any pregnant woman who expresses her worries can undergo such a test. Ultimately, this line of reasoning would also be valid for the introduction of RCS.

In cases of known genetic predisposition with an increased risk of monogenic disease in the child, *invasive prenatal diagnostics* (chorionic villus sampling, amniocentesis) are currently offered during pregnancy and are financed by health insurance companies. To avoid the birth of an affected child, the only option is to terminate the pregnancy after abnormal prenatal findings.

Alternatively, *preimplantation genetic testing (PGT)* can be offered to avoid the birth or pregnancy of an affected child. This procedure is carried out at specialist centres, requires extracorporal techniques via medically assisted reproduction and is only permitted if there is a high risk of the child developing a serious illness due to genetic predisposition. Genetic testing is carried out on embryonic cells just a few days after fertilisation, on day 3 (blastomeres) or days 5 – 7 (trophectoderm). In Germany, the couple’s application for authorisation to undergo PGT is assessed by an interdisciplinary ethics committee. Thus, PGT is subject to regulation by the medical associations in the individual federal states. *The option of gamete donation* in German-speaking countries often results in medical travels across borders. The use of donor sperm is permitted in Germany, Austria and Switzerland under certain legal conditions, whereas egg donation is not (yet) permitted in Germany and Switzerland, unlike in Austria.

## The ethical perspective

The Ethics Committee of the European Society of Human Reproduction and Embryology (ESHRE) has reflected on and provided ethical guidance for a responsible implementation of preconception carrier screening in the context of assisted reproduction [19]. Possible harms and disadvantages were divided into socio-cultural harms (disability rights and medicalization critique, objection in terms of eugenics) and individual harms (reproductive dilemmas, impact of health risks for carriers, and stigmatization and discrimination).

The following arguments have to be considered:

### Reproductive autonomy

When couples seek RCS *before pregnancy*, the quality of their decision and the selection of reproductive options improve compared to couples who only become aware of a serious condition in their child *during pregnancy* or after birth. When providing genetic counselling to parents of affected children, the attending physicians are often confronted with the following statement: “If only we had known this before!”

### Proportionality

In our society it is firmly established that invasive prenatal diagnostics are permitted as a statutory health insurance benefit e.g. for a 35-year-old woman to rule out a chromosomal disorder (risk approx. 0.5 %). Following this logic, it is disproportionate not to introduce a test that would identify 4 – 5 % of couples who have a high risk of 25 % for a serious disease in their children. In a recent Italian study on cost analysis of RCS, it was pointed out that the conditions tested by the carrier screening, when accounted as a group, have a higher incidence compared with the aneuploidies screened prenatally in the Italian population. The authors conclude that this aspect should be taken into consideration in order to guarantee an equitable and coherent offer of prenatal genetic screening [20].

### Justice

An important fairness argument in favour of an RCS is that in Germany it is already possible to determine a carrier status among the data retrieved by exome or genome analysis regardless of the primary indication. It is therefore socially unjust that exome or genome analyses can be carried out at the expense of the health care system and that additional carrier status information in the sense of an RCS can be communicated on request, which couples who wish to have children without a corresponding reason – but with the same risk of being carriers – do not receive.

In the debate on justice, it should not be overlooked that, given the scarcity of resources for healthcare, a cost-benefit analysis should also be permissible, as it has been done in other European countries such as Italy [20]. In the Italian study, costs were calculated according to the ACMG tiered approach. From a universal health care system perspective, all the 3 ACMG tiers considered in the study would be more cost-effective than no screening.

### Stigmatization of disability

Despite the widespread use of prenatal testing for decades, the introduction of new technologies has always been accompanied by arguments for reproductive autonomy on the one hand and warnings against selection, stigmatization, and discrimination against people on the other [21]. These positions have a long-standing history in Germany. However, other democratic countries have shown how to overcome political differences between stakeholders for the benefit of patients.

**Figure 1:**
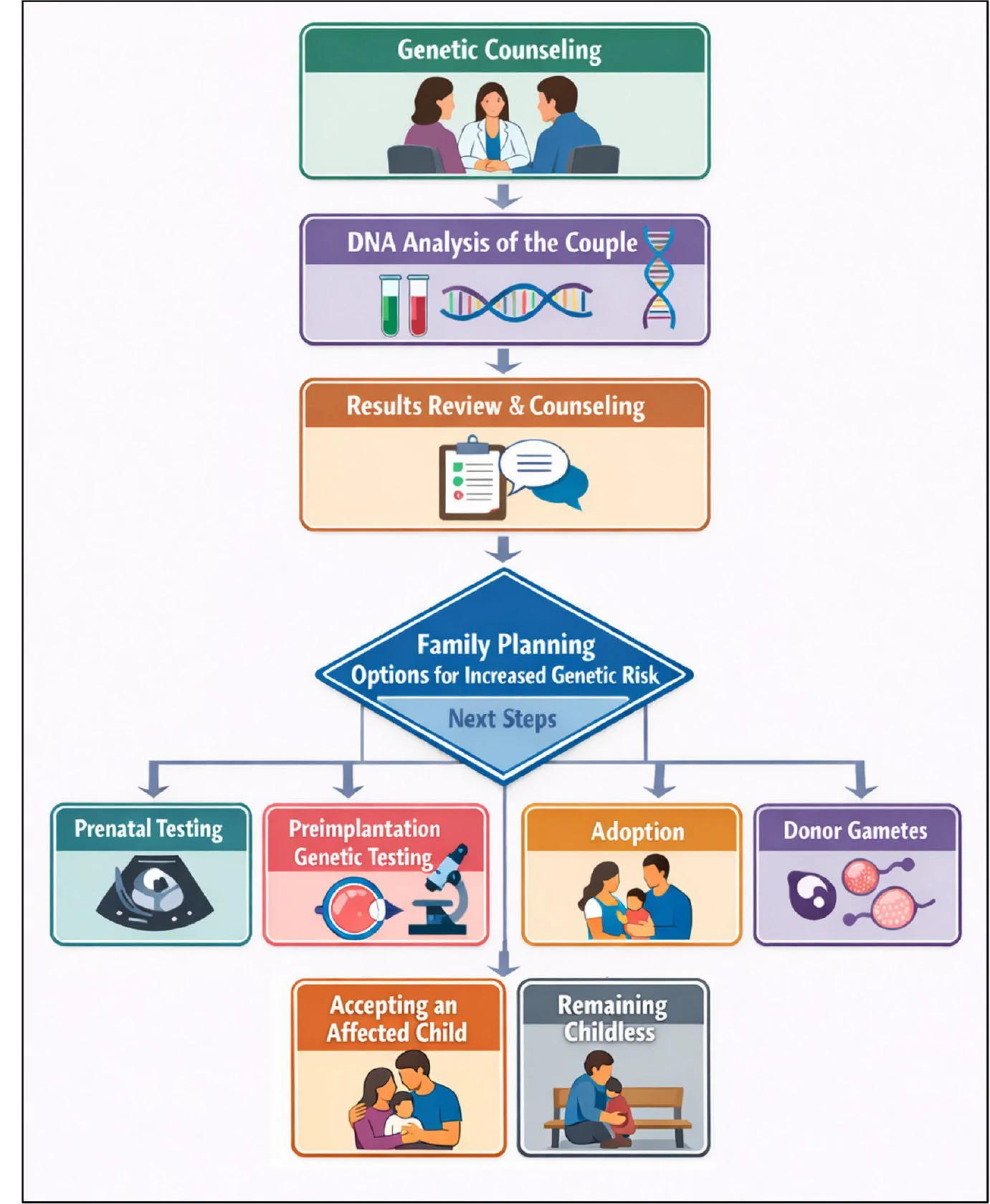
Flowchart of reproductive carrier screening.

## Conclusion

It is difficult to put forward perspectives for the implementation of an RCS for discussion in a society where there is still a controversial debate about abortion or PGT as an achievement of reproductive autonomy. To quote the Ethics Committee of the Giordano Bruno Foundation [22], which commented on the issue in the context of the approval of PGT: “Anyone who takes a rational, humanistic view should realize that disabled and sick people deserve our full support, but disability and illness do not.” And further: “If parents decide against a genetic defect, their motive is to avoid burdens on their future child, to give them the best possible start in life, and to spare themselves additional hardship. There is nothing reprehensible about this” [22].

We believe that a broad social and political debate on RCS is necessary in Germany, given the rapid developments in genetic and reproductive medicine.
